# A Case of Nasal Glial Heterotopia That Can Be Misdiagnosed as Storiform Patterned Sclerotic Fibroma/Collagenoma

**DOI:** 10.5146/tjpath.2023.13053

**Published:** 2024-05-18

**Authors:** Fatma Gundogdu, Deniz Ates Ozdemir, Ibrahim Vargel

**Affiliations:** Department of Pathology, Hacettepe University, Faculty of Medicine, Ankara, Turkey; Department of Plastic Reconstructive and Aesthetic Surgery, Hacettepe University, Faculty of Medicine, Ankara, Turkey

**Keywords:** Nasal dorsum, Glial heterotopia, Sclerotic fibroma/collagenoma, Child, Case-report

## Abstract

**Objective:** Nasal glioma, also known as nasal glial heterotopia, is a rare tumor-like lesion that often affects newborns or infants with no hereditary predisposition.

**Case Report:** A 4-year-old child with a growth on the nasal dorsum since birth was diagnosed with nasal glial heterotopia/nasal glioma. The lesion showed a sclerotic fibroma/collagenoma-like storiform pattern with entrapped glial tissue that was S100 and GFAP positive.

**Conclusion:** When a biopsy of the nasal dorsum demonstrates sclerotic microscopic findings with a storiform pattern, nasal glioma should be considered before making a diagnosis in the collagen-rich tissue spectrum (collagenoma or Gardner's fibroma), and an immunohistochemical panel should be requested to demonstrate the presence of an unrecognized light microscopically visible glial component.

## INTRODUCTION

Nasal glioma, also known as nasal glial heterotopia, is an uncommon developmental abnormality that usually appears at birth or a few years later. It can, however, be noticed in people of all ages, including adults. There is no known genetic susceptibility. These lesions have no connection to the central nervous system (CNS). Gliomas of the nose can be extranasal (60%), intranasal (30%), or mixed (10%). Extranasal forms are most common on the nasal dorsum, whereas intranasal forms are most common on the lateral nasal wall of the middle turbinate. The clinical presentation varies depending on the location and is not specific. Clinical symptoms frequently include nasal obstruction, nasal polyps, chronic sinusitis, and chronic otitis media. The treatment for nasal glial heterotopia is total excision. If the resection is inadequate, recurrence can occur (1).

Nasal gliomas are made up of mature glial tissue that is mixed with fibrous and vascularized stroma and inflammatory cells. There may be gemistocytic astrocytes, neuronal cells, and, less typically, ependymal cells distributed throughout. The degree of fibrosis and calcification varies. Glial tissue may be difficult to detect in some situations due to thick inflammatory cells or severe fibrosis/sclerosis, particularly in long-standing and adult cases. S100 and GFAP (1–3) are immunohistochemically positive in heterotopic glial tissue.

In this report, we will demonstrate a case of nasal glioma with collagenous fibroma-like storiform collagen, an unusual histological feature that has not previously been discussed. In this instance, we wanted to emphasize that when dense collagen with storiform morphology is identified in a pediatric patient’s dorsum of the nose, nasal glioma should be added to the differential diagnostic spectrum even if the glial tissue is not visible at the H&E level.

## CASE PRESENTATION

**Figure 1:**
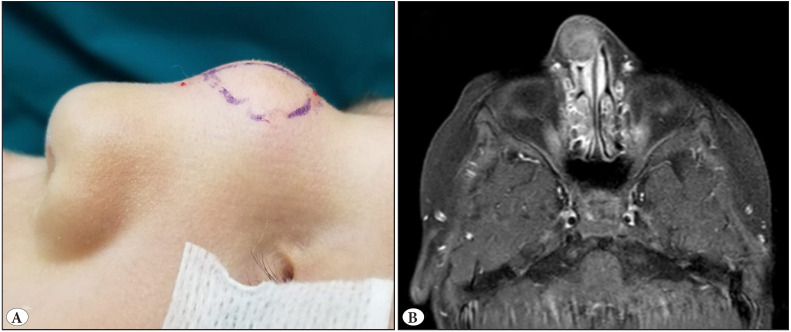
**A)** The external physical examination revealed a 2 cm-sized mass on the nasal dorsum. **B)** T1-weighted MRI shows a 2.2x1.9x1.6 cm-sized, hypointense, well-circumscribed, nodular solid mass on the tip of the nose.

The patient is a 4-year-old male who has had a nasal dorsum mass since birth. He was admitted to another hospital four years ago with a complaint of nasal edema and was treated with beta-blockers for five months after being diagnosed with hemangioma. However, as the swelling persisted, the patient was admitted to our hospital for additional testing. A 2x2 cm firm, palpable lump on the nasal dorsum was identified during the physical examination (Figure 1). There were no obvious signs of nasal blockage, and the intranasal cavity was unremarkable in the fiberoptic examination. On magnetic resonance imaging, a 2.2x1.9x1.6 cm-sized, well-circumscribed solid nodular lesion that was hypointense in T1 and T2A was seen on the tip of the nose and extended to the right alar wing (Figure 1). As a hemangioma, there was no signaling or flow void, and no relationship with the central nervous system was observed. As a result, hemangioma and nasal encephalocele had been ruled out radiologically. During the follow-up, the patient was closely monitored until the entire mass excision area was stable.

**Figure 2:**
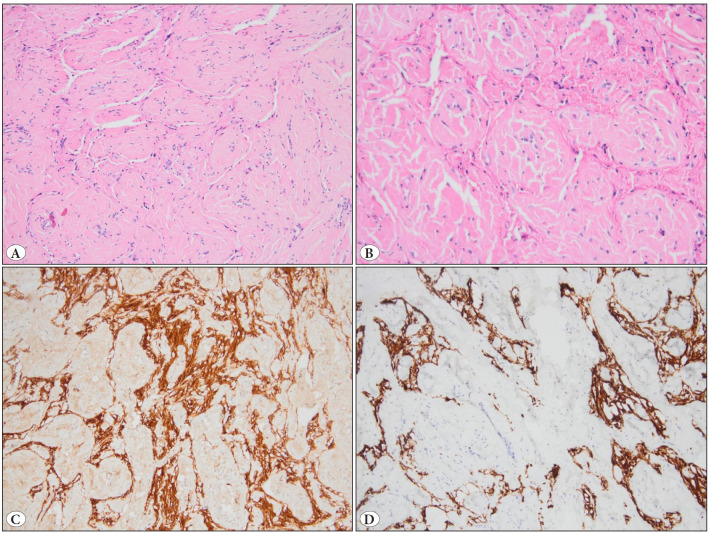
In the histopathological evaluation of the specimen, **A)** low power magnification shows a highly fibrotic hypocellular lesion (H&E 100x), **B)** high power magnification shows the lesion is composed of spindle cells and collagen bundles arranged in a fascicular and whorled pattern, and no neuropil or ganglion cells can be seen (H&E 200x). The glial tissue that was entrapped with fibrovascular stroma was positive for **C)** S100 and **D)** GFAP.

The macroscopic examination of the excision material revealed a firm, white, nodular 1.6x1.3x0.8 cm lesion. Microscopically, a highly fibrotic hypocellular lesion was composed primarily of spindle cells and collagen bundles organized in a fascicular pattern (Figure 2). There were no apparent neuropil or ganglion cells with H&E (Figure 2). The glial tissue entrapped by a fibrovascular stroma was immunohistochemically positive for S100 (Figure 2) and GFAP (Figure 2), but negative for CD34 and EMA. Neu-N immunostaining revealed no neuronal cells. These findings led to the diagnosis of nasal glial heterotopia/nasal glioma.

## DISCUSSION

Nasal glioma, also known as nasal glial heterotopia, is a rare, non-neoplastic lesion that frequently develops in newborns or babies who do not have a familial susceptibility. It can also be seen in adults in a more sclerotic form; the diagnosis is challenging. The literature has roughly 300 cases, with a frequency of one in every 20.000-40.000 live births and a female predominance. Based on their location, nasal glial heterotopias are categorized as extranasal, intranasal, or mixed lesions. Extranasal lesions are the most common kind and are typically found on the dorsum of the nose (3). Since the early 1900s, “nasal glioma” has been used to describe lesions of mature glial tissue on the dorsum of the nose. However, because it has been demonstrated that these lesions are not real neoplasms but rather developmental anomalies, they are now referred to as nasal glial heterotopia (4).

The histological appearances of nasal glial heterotopia range from a large island of glial tissue with neuropil and large, sometimes multinucleated astrocytes that is visible in H&E to extensive fibrotic/sclerotic forms that mask the glial tissue that is only visible with immunohistochemical stains like S100 and GFAP, as seen in our cases. This type of severe fibrosis is typically observed in older patients, most of whom are adults. Despite the fact that our patient is a 4-year-old child, sclerosis is a dominant pattern. Neuronal, ependymal, and leptomeningeal cells can be seen. In some situations, calcification or cystic degeneration is also observed. While Neu-N aids in the identification of neuronal cells, SSTR2A can be utilized to confirm the existence of leptomeningeal cells (1,2,4).

The differential diagnosis of midline nasal region lesions includes nasal glial heterotopia, nasal encephalocele, nasal dermoid, hemangioma, and lipoma. The distinction between nasal glial heterotopia and nasal encephalocele is essential because of the risk of intracranial spreading of infections in an encephalocele and causing complications like meningitis. However, there are no pathological criteria to distinguish these two lesions. Only the clinical or radiological findings that showed a CNS connection led to a diagnosis of encephalocele over heterotopia (4). Lately, molecular studies have shown that these two lesions share some molecular alterations, like copy number variations in chromosomes 16, 17, and 19, and supported the idea that these lesions are part of the same spectrum (2). In our case, radiological examinations showed no relationship between the central nervous system and the lesion. A nasal encephalocele diagnosis was therefore excluded. 

The microscopic examination of our patient showed significant fibrosis that obscured glial tissue. It was revealed immunohistochemically with S100 and GFAP. In cases like this, diagnosing nasal glial heterotopia may be challenging if this differential diagnosis is not considered. In a highly collagenous lesion like this, we might also think of storiform collagenoma or sclerotic fibromas (like Gardner fibroma) in the differential diagnosis, besides other common differentials. 

Circumscribed storiform collagenoma, or sclerotic fibroma, is a rare, well-circumscribed hypocellular dermal tumor composed of thick collagen bundles and uniform spindle cells arranged in a whorled pattern. Immunohistochemically, CD34 and Factor VIIIa can be positive. It can be seen sporadically and is associated with Cowden syndrome (5). Gardner fibroma is a benign soft tissue lesion seen in any part of the body, whether located superficially or deeply. They mainly occur in children and adolescents and are associated with familial adenomatous polyposis (FAP) and Gardner syndrome. Histologically, they are composed of thick, haphazardly arranged collagen bundles and spindled fibroblastic cells without atypia infiltrating the adipose tissue which characteristically shows nuclear Beta-catenin expression in most cases (6).

Finally, when a sclerotic lesion in a storiform pattern is detected in a pediatric patient, especially in the nasal localization, a nasal glial heterotopia should be considered before a diagnosis of collagen-rich lesions (collagenoma or Gardner’s fibroma) is made at the H&E level. Even if neuropil is not visible in H&E sections, the presence or absence of glial tissue should be evaluated.

## Conflict of Interest

The authors declare that they have no conflict of interest for this article.

## Funding

None.

## Informed Consent

The patient or their guardians consented for publication
